# Determinants of unintended pregnancies among currently married women in Uganda

**DOI:** 10.1186/s41043-020-00218-7

**Published:** 2020-12-07

**Authors:** Ronald Wasswa, Allen Kabagenyi, Leonard Atuhaire

**Affiliations:** 1 Department of Statistical Methods and Actuarial Science, School of Statistics & Planning, College of Business and Management Sciences, Makerere University, P.O. Box 7062, Kampala, Uganda; 2 Department of Population Studies, School of Statistics & Planning, College of Business and Management Sciences, Makerere University, P.O. Box 7062, Kampala, Uganda; 3 Department of Planning and Applied Statistics, School of Statistics & Planning, College of Business and Management Sciences, Makerere University, P.O. Box 7062, Kampala, Uganda

**Keywords:** Unintended pregnancies, married women, Uganda

## Abstract

**Background:**

Unintended pregnancies are no longer bound to teenagers or school-going children, married women in Uganda, as well do experience such pregnancies though little has been investigated on them. This study examines the determinants of unintended pregnancies among currently married women in Uganda.

**Methods:**

In this study, we used data from the 2016 Uganda Demographic and Health Survey (UDHS) which comprised of 10,958 married women aged 15–49 years who have ever been pregnant. The analysis was done using descriptive analysis, logistic regression, and the generalized structural equation model.

**Results:**

The study showed that 37% of pregnancies among married women were unintended. Young women, living in poor households, staying in rural areas, women in the Eastern and Northern region, Muslim women, lack of knowledge on ovulation period, discontinuation of contraceptives, non-use of and intention for contraceptives, high age at sexual debut, high age at first birth, and high parity were directly associated with a higher risk of unintended pregnancies. Relatedly, discontinuation of contraceptives regardless of the place of residence, region, woman’s age, education, household wealth, access to family planning messages were associated with higher odds of unintended pregnancies. Older women and those in rural areas who had more children were also at a higher risk of similar pregnancies. However, having more children while using contraceptives, being educated, living in a wealthier household, and having access to family planning messages significantly lowered the risk of unintended pregnancies.

**Conclusion:**

Increased access to family planning messages, empowering women as well as having improved household incomes are key preventive measures of unintended pregnancies. There is a need to provide quality contraceptive counseling through outreaches so that women are informed about the different contraceptive methods and the possible side effects. Having a variety of contraceptive methods to choose from and making them accessible and affordable will also encourage women to make informed choices and reduce contraceptive discontinuation. All these coupled together will help women have their desired family sizes, increase the uptake of contraceptives and significantly reduce unintended pregnancies.

## Background

Unintended pregnancies are pregnancies that are either unwanted or mistimed at the time of conception [1]. Globally, an estimated 9817 women become pregnant every day without planning [1]. These pregnancies have brought a public health concern in both developed and developing countries because of their association with adverse social, health, and economic outcomes for both mothers and their children [2]. Not only in Africa, studies in the USA, China, Netherlands, and France have also registered increasing trends in unplanned births [3–6]. However, 8 in 100 women experience unplanned pregnancies in Africa, the highest rate globally with eastern Africa taking the lead [5].

In Uganda, the situation is also alarming with an estimate of 1.2 million unintended pregnancies being registered in 2008 representing more than half of the country’s 2.2 million pregnancies [6] Since then, the prevalence has seemingly remained high with 52% of such pregnancies registered in 2013 [7] leading to many deaths and abortions [8]. Other than mortality and abortion, studies also show that women who experience unintended pregnancies are exposed to health-related problems [9, 10] such as like hypertension, hypothyroidism, diabetes, hepatitis, and cardiac disorders [11], have low psychosocial well-being and high maternal depression [12–14]. In Nepal, women with such pregnancies still spend a lot of money to take care of their pregnancies [14]. However, it should be noted that this expenditure is not only inclined on these women but also to the whole nation as well. Sonfield and colleagues showed that a big proportion of a country’s expenditure caters to unintended pregnancies which negatively affects the growth of the nation and the population as a whole [15]. Other consequences include inadequate prenatal care, immunization, antenatal care, breastfeeding, and parenting [16–19].

According to 2016 UDHS, the total wanted fertility rate among Ugandan women is five children as compared with the actual total fertility rate of six children; implying that women in Uganda are having one child more than they want [20]. Similarly, the contraceptive prevalence rate among married women of reproductive age in Uganda is still very low with 3 in 10 wishing to delay or avoid pregnancy but are not using any contraceptive measure and yet desire a small family size [20]. Therefore, the combination of low contraceptive use and smaller desired family size among married women implies high levels of unmet need for family planning which ranks Uganda highly in Sub-Saharan Africa [20, 21]. However, relatively very little research on unintended pregnancy among married women has been documented in Uganda though studies have done elsewhere in countries including Ethiopia, Bangladeshi, Nigeria, Kenya, Malawi, and Ivory Coast [22–27]. However, most of these studies were limited in providing an understanding of the inter-relationships among the factors associated with unintended pregnancies since researchers used binary regression models. This study therefore sought to address the shortfalls in regard to scope and methodology in assessing the determinants of unintended pregnancy among currently married women in Uganda by answering the following objectives: (1) Identify the direct factors influencing unintended pregnancies. (2) Identify the indirect factors associated with unintended pregnancies among married women in Uganda.

## Data and methods

**Fig. 1:**
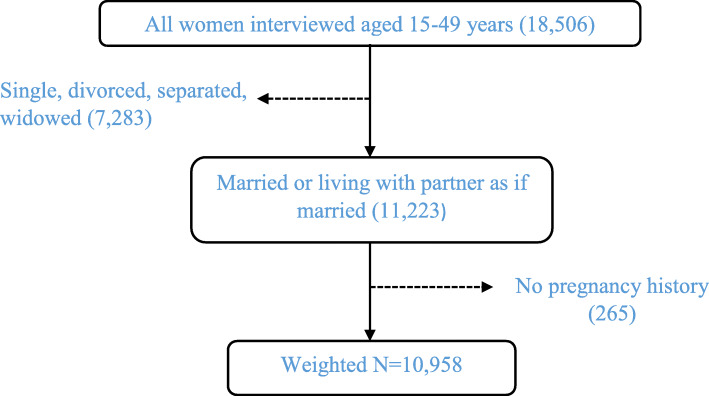
Sample selection procedure

Data for this study was based on secondary data from the 2016 Uganda Demographic and Health Survey (UDHS). The authorization to use the data was obtained from Measure DHS by providing a description of the study through their website. The 2016 UDHS was a national representative survey that employed a two-stage stratified sample design. In the first stage, 696 enumeration areas were selected from a list of clusters based on the 2014 Uganda Population and Housing Census sample frame. The second stage involved a systematic sampling of 20,880 households within each cluster from which all women of child-bearing age (15–49 years), who were either permanent residents of the households or visitors who slept in the households the night before the survey were eligible to be interviewed. A total of 18,506 women aged 15–49 years were interviewed. In this study, we started by excluding all women who were never married as well as those who were divorced, separated, and widowed. This was followed by excluding women who had no pregnancy history. Given that DHS captures the birth histories of women, those who had never given birth were not currently pregnant and had never had any terminated pregnancy at the time of the survey were also excluded in the sample selection. Overall, a weighted total of 10,958 women aged 15–49 years formed our study sample. Figure 1 below shows the derivation of the sample.

### Variables and their measurements

In this study, unintended pregnancy was the dependent variable. In the 2016 UDHS, unintended pregnancy, Y_1_, was measured by the question, “At the time you became pregnant, did you want to become pregnant then, did you want to wait until later, or did you not want to have any (more) children at all?” [20]. Women were asked this question for all the last five children produced before the time of the survey. Based on this definition, we generated unintended pregnancy as a binary outcome coded 1 if any of the pregnancies was unintended (if any of the pregnancies occurred at a time when the woman would have wanted it later or did not want it at all) and 0 otherwise (if all the pregnancies occurred at a time when the woman wanted them).

Predictor variables considered included woman’s age, X_1_ (15–24, 25–34, and 35–49); wealth index, X_2_ (poor, middle, rich); woman’s education level, X_3_ (no education, primary, secondary/higher); place of residence, X_4_ (urban, rural); region, X_5_ (Central, Eastern, Western and Northern); religion, X_6_ (Catholic, Protestant, Muslims, Others; where other religions include Seventh Day Adventists (SDA), Orthodox, Born again/Pentecostal/Evangelical, Baha’i, Baptist, Presbyterian, Jehovah’s witness, Salvation army, Traditionalists and other unknown religions); Occupation, X_7_ (not working, professional/technical/managerial/clerical, agricultural/household and domestic, Sales/services, and manual), access to family planning messages through media, X_8_ (this was measured using four questions that required whether a woman was exposed to family planning messages on radio, television, newspapers, and phone in the last month. All the responses to these questions were merged and coded as zero for no exposure to all the media and one for those who heard FP messages from any of the four sources); husband’s age, X_9_ (15–24, 25–34, 35–44 and 45+); partner’s level of education, X_10_ (no education, primary, secondary/higher). Additional variables included: age at sexual debut, Y_2_ (continuous), age at first marriage, Y_3_ (continuous), age at first birth, Y_4_ (continuous), total children ever born, Y_5_ (count), knowledge on ovulation, Y_6_ (during/after period, middle of the cycle, before period begins and do not know following the DHS categorization). Those who did not know included those who said that ovulation occurs at any time, those who did not know anything and any other category; contraceptive use and intention, Y_7_ (in the 2016 UDHS, women were asked if they were using any contraceptive method and for those who were not using were asked whether they would use in future). Contraceptive methods included male or female sterilization, injectable, intrauterine devices, contraceptive pills, implants, female or male condoms, standard days method, lactational amenorrhoea, emergency contraception use of rhythm, periodic abstinence, withdrawal, and folk methods [20]. This was the basis for categorization with 1 for those who were using any method and non-users who intend to use later while 0 for those who do not intend to use. Discontinuation of contraceptives, Y_8_: This was obtained from the contraceptive calendar that traces the use and reasons for discontinuation of contraceptives among women. This study considered women whose reason for discontinuation was to become pregnant not to have actively discontinued. Therefore, this was a binary variable with married women who were using contraceptives and those whose reason for discontinuation was to become pregnant coded 0 while those who discontinued because they had become pregnant (contraceptive failure), husband disapproval, side effects, costs/ access of methods, inconvenience to use, and others reasons were considered to have perfectly discontinued and coded 1.

In order to have the inter-relationship among these variables, we grouped the variables into two categories that are exogenous and endogenous variables. An exogenous variable is a factor in a model whose value is independent of the state of other variables in the system while an endogenous variable on the other hand is one which is influenced by one or more independent variables (excluding itself). The selected exogenous variables were women’s age, place of residence, region of residence, wealth index, woman’s education level, husband’s education level, husband’s age, occupation, religion, and access to family planning (FP) messages through media. On the other hand, there were eight endogenous variables such as age at first marriage, age at first sex, age at first birth, children ever born, contraceptive use and intention, discontinuation of contraceptives, and unintended pregnancy. In Uganda, not only age at first sex being much lower than the age at first marriage but also the rate of child marriage is also high [20]. Therefore, a situation where age at marriage is low tends to result in early sex initiation, early childbearing, lack of knowledge on fertility including underuse of contraceptives. It is anticipated that this later results in higher fertility and unintended pregnancy. This formed the basis of identifying endogenous variables for the study.

### Data analysis

Data analysis was done using STATA 15 at three stages. The data were first weighted to ensure the representativeness of the sampled data as required for DHS data [20]. A weighting variable generated using the sample weight variable in the DHS data was applied in all statistical commands. At the first stage of analysis, a descriptive summary (either as percentages for the categorical variables or mean for the continuous variables) of the factors was done. In the second stage, the determinants of unintended pregnancy were assessed by the selected characteristics using a bivariate logistic regression model. The results at this stage indicated how the exogenous variables independently influenced the endogenous variables. The purpose of this level was to identify variables for further analysis at the multivariate level.

**Fig. 2:**
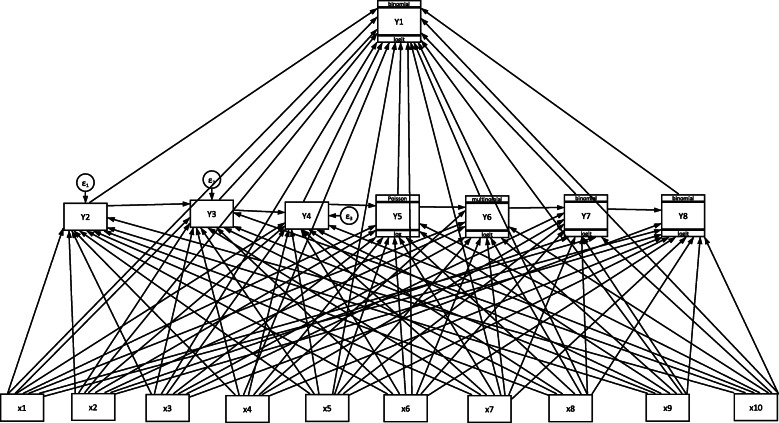
The GSEM model showing the selected exogenous and endogenous variables

In the third stage, the net-impact of the exogenous variables on the endogenous variables were established using a Generalized Structural Equation Model (GSEM). The GSEM was appropriate and it has been used and applied in health-related studies [28, 29]. Generalized structural equation models are a family of statistical models that seek to assess and explain the relationship between multiple independent variables and outcome variables in the same model even if outcome variables are varied in nature (continuous, dichotomous, ordinal and count, among others). Therefore, several multiple relationships of endogenous and exogenous variables were investigated using path analysis as shown in Fig. 2 below. The relationships comprise of direct and indirect effects on the endogenous factors.

The model was based on the following equations:

1 $$ \ln \left(\frac{\mathrm{P}\left({\mathrm{Y}}_1=1\right)}{1-\mathrm{P}\left({\mathrm{Y}}_1=1\right)}\right)={\beta}_0+{\sum}_{j=2}^8{\beta}_{1j}{Y}_j+{\sum}_{j=1}^{10}{\beta}_{1j}{X}_j $$

2 $$ {\mathrm{Y}}_2={\beta}_1+{\sum}_{j=1}^{10}{\beta}_{2j}{X}_j+{\varepsilon}_1 $$

3 $$ {\mathrm{Y}}_3={\beta}_2+{\beta}_{32}{Y}_2+{\sum}_{j=1}^{10}{\beta}_{3j}{X}_j+{\varepsilon}_2 $$

4 $$ {\mathrm{Y}}_4={\beta}_3+{\sum}_{j=2}^3{\beta}_{4j}{Y}_j+{\sum}_{j=1}^{10}{\beta}_{4j}{X}_j+{\varepsilon}_3 $$

5 $$ {Y}_5={e}^{\beta_4+{\sum}_{j=2}^4{\beta}_{5j}{Y}_j+{\sum}_{j=1}^{10}{\beta}_{5j}{X}_j} $$

6 $$ \mathrm{In}\left(\frac{\mathrm{P}\left({\mathrm{Y}}_6={y}_k\right)}{1-\mathrm{P}\left({\mathrm{Y}}_6={y}_k\right)}\right)={\beta}_5+{\sum}_{j=2}^5{\beta}_{6j}{Y}_j+{\sum}_{j=1}^{10}{\beta}_{6j}{X}_j $$

7 $$ \ln \left(\frac{\mathrm{P}\left({\mathrm{Y}}_7=1\right)}{1-\mathrm{P}\left({\mathrm{Y}}_7=1\right)}\right)={\beta}_6+{\sum}_{j=2}^6{\beta}_{7j}{Y}_j+{\sum}_{j=1}^{10}{\beta}_{7j}{X}_j $$

8 $$ \ln \left(\frac{\mathrm{P}\left({\mathrm{Y}}_8=1\right)}{1-\mathrm{P}\left({\mathrm{Y}}_8=1\right)}\right)={\beta}_7+{\sum}_{j=2}^7{\beta}_{8j}{Y}_j+{\sum}_{j=1}^{10}{\beta}_{8j}{X}_j $$

Where *P*(*Y*_*j*_) with *j* = 1, 7, 8 is the probability that a woman had an unintended pregnancy, was using or had the intention of contraceptives and discontinued contraceptives; *β*_0_, …, *β*_7_ are the intercepts; *β*_1*j*_, …, *β*_8*j*_ are regression coefficients; *X*_*j*_ are explanatory variables; *Y*_5_ is the expected number of children per woman; *e* is the base of natural logarithms; *Y*_2_, *Y*_3_, *Y*_4_ is the woman’s age at sexual debut, age at first marriage, and age at first birth respectively with *ε*_*i*_ being the error terms; *y*_*k*_ are the unordered outcomes of knowledge on ovulation.

We ensured that all the selected variables were independent, and the continuous variables met the assumptions of normality [30, 31]. The variance inflation factor (VIF) was also performed to detect any multi-collinearity between the explanatory variables. None of the factors showed multi-collinearity problems.

A diagnostic test was performed by running three models in GSEM in order to check the level of adequacy of the explanatory variables in predicting the outcome variable by using the likelihood ratio test, the Akaike Information Criterion (AIC) and the Bayesian Information Criterion (BIC). Model 1 controlled for woman’s demographic characteristics, model 2 controlled for woman’s socio-economic and husband’s characteristics while model 3 controlled for all the demographic, socio-economic, and intermediate factors. Insignificant paths were also removed from the final model in order to maximize its efficiency unless if there was strong evidence of association from the previous studies.

## Results

Table 1 presents the selected demographic, socio-economic, and intermediate characteristics of currently married women aged 15–49 years. The results indicate that majority of married women were aged 25–34 years (40%), Catholics (40%), and three quarters staying in rural areas. Further, a great number were also from the eastern region of Uganda (28%). The results also show that the majority of married women were employed in agricultural/household and domestic work (46%) while the highest number had attained primary education (59%). Still, 2 in 5 women were living in poor households, 70% could access family planning messages through media while 16% did not know anything about ovulation. Additionally, 22% of the married women were neither using contraceptives nor had the intention for the future, 25% had discontinued the use of contraceptives, and 37% of the pregnancies were unintended. Still, the average age at first marriage, age at sexual debut, and age at first birth among married women were respectively 18, 17, and 19 years giving birth on average four children. Additionally, 36% of the women were married to husbands aged 25–34 years and more than half of the husbands having attained at least a primary level of education.

**Table 1:** Distribution of married women by the selected variables

Categorical variables	Frequency (*N* = 10,958)	Percentage (%)
Woman’s age		
15–24	3041	27.8
25–34	4343	39.6
35–49	3574	32.6
Wealth index		
Poor	4273	39.0
Middle	2143	19.6
Rich	4542	41.4
Woman’s education level		
No education	1339	12.2
Primary	6509	59.4
Secondary/higher	3110	28.4
Place of residence		
Urban	2561	23.4
Rural	8397	76.6
Region		
Central	2931	26.8
Eastern	3020	27.6
Western	2842	25.9
Northern	2165	19.7
Religion		
Catholic	4367	39.8
Anglican	3427	31.3
Muslim	1454	13.3
Others	1710	15.6
Occupation		
Not working	1747	16.0
Professional/technical/managerial/ clerical	971	8.9
Agricultural/household and domestic	5065	46.2
Sales/services	1516	13.8
Manual	1659	15.1
Access to family planning messages		
No	3264	29.8
Yes	7694	70.2
Husband’s age		
≤ 24	1049	9.6
25-34	3888	35.5
35-44	3366	30.7
45 and above	2655	24.2
Husband’s education level		
No education	703	6.4
Primary	5,708	52.1
Secondary/Higher	4241	38.7
Do not know	306	2.8
Knowledge on ovulation		
During/after period	5569	50.8
Middle of the cycle	2593	23.7
Before period begins	998	9.1
Do not know	1798	16.4
Contraceptive use and intention		
No	8537	77.9
Yes	2421	22.1
Discontinuation of contraceptives		
No	2737	25.0
Yes	8221	75.0
Unintended pregnancy		
No	6856	62.6
Yes	4102	37.4
**Continuous and count variables**	**Mean**	**SD**
Age at sexual debut	16.5	2.6
Age at first marriage	18.4	4.1
Age at first birth	18.7	3.3
Total children ever born	4.2	2.8

SD is the standard deviation; all estimates are based on weighted data

### Association of unintended pregnancy with the selected factors

Results in Table 2 show the bivariate analysis of the factors associated with unintended pregnancies. Woman’s age, wealth index, woman’s education level, place of residence, region, religion, occupation, access to family planning messages through media, husband’s age, husband’s education level, knowledge on ovulation, age at sexual debut, age at first marriage, age at first birth, total children ever born, knowledge on ovulation, discontinuation of contraception, and contraceptive use or intention were important factors in explaining unintended pregnancies. More specifically, older women, the rich, those from the western region, women employed in professional/technical/managerial/clerical sector, those who could access family planning messages through media, those married to older men, women who knew that ovulation occurs in the middle of the cycle, those with higher age at sexual debut, higher age at first marriage, higher age at birth and women who were using contraceptives or had the intention of contraceptives were less likely to have unintended pregnancies. Women who were associated with increased odds of unintended pregnancies were those residing in rural areas, with primary education, Moslems, located in Eastern and Northern regions, employed in agricultural/household and domestic work, had educated husbands, high parity, without knowledge on ovulation and had discontinued use of contraceptives.

**Table 2:** Bivariate association between unintended pregnancies and the selected factors

Selected characteristics	OR(95%CI)
Woman’s age	
15–24^†^	1.00
25–34	0.81(0.73**–**0.89)**
35–49	0.50(0.45**–**0.56)**
Wealth index	
Poor^†^	1.00
Middle	0.88(0.79**–**0.98)*
Rich	0.53(0.48**–**0.58)**
Woman’s education level	
No education^†^	1.00
Primary	1.59(1.40**–**1.80)**
Secondary/higher	1.02(0.89**–**1.17)
Place of residence	
Urban^†^	1.00
Rural	1.67(1.52**–**1.84)**
Region	
Central^†^	1.00
Eastern	1.69(1.52**–**1.87)**
Western	0.84(0.75-0.94)**
Northern	1.60(1.43-1.80)**
Religion	
Catholic^†^	1.00
Anglican	1.06(0.97**–**1.17)
Muslim	1.34(1.19**–**1.51)**
Others	1.14(1.02**–**1.28)*
Occupation	
Not working^†^	1.00
Professional/technical/managerial/clerical	0.64(0.54**–**0.76)**
Agricultural/household and domestic	1.19(1.07**–**1.33)**
Sales/services	0.87(0.75**–**1.00)
Manual	0.97(0.84**–**1.11)
Access to family planning messages	
No^†^	1.00
Yes	0.90(0.82**–**0.98)*
Husband’s age	
≤24^†^	1.00
25–34	1.03(0.89**–**1.18)
35–44	0.85(0.73**–**0.97)*
45 and above	0.54(0.47**–**0.63)**
Husband’s education level	
No education^†^	1.00
Primary	1.73(1.46**–**2.06)**
Secondary/Higher	1.33(1.11**–**1.58)**
Do not know	1.30(0.98**–**1.74)
Knowledge on ovulation	
During/after period^†^	1.00
Middle of the cycle	0.87(0.79**–**0.95)**
Before period begins	1.06(0.93**–**1.22)
Do not know	1.12(1.01**–**1.25)*
Age at sexual debut	0.48(0.37**–**0.62)**
Age at first marriage	0.43(0.35**–**0.52)**
Age at first birth	0.37(0.29**–**0.46)**
Total children ever born	1.08(1.06**–**1.09)**
Contraceptive use and intention	
No^†^	1.00
Yes	0.40(0.36**–**0.44)**
Discontinuation of contraceptives	
No^†^	1.00
Yes	1.97(1.81**–**2.15)**

† is a reference category; OR is the odds ratio, 95% CI is the confidence interval, *p < 0.05, **p < 0.01, the assessment was based on a bivariate logistic regression model with χ^2^=0.000

### Direct determinants of unintended pregnancies among married women

After adjusting and controlling for possible confounders, results in Table 3 show that women aged 25–34 (AOR = 0.37, 95% CI = 0.33–0.43) and those aged 35–49 (AOR = 0.12, 95% CI = 0.09–0.15) were less likely to experience unintended pregnancy compared to those less than 25 years. Similarly, married women living in rich households (AOR = 0.72, 95% CI = 0.64-0.82), those who were using contraceptives or were non-users but had intentions of contraceptive use (AOR = 0.47, 95% CI = 0.42–0.54), women from the western region (AOR = 0.72, 95% CI = 0.63–0.82), those whose husbands were aged 35–44 years (AOR = 0.73, 95% CI = 0.60–0.89) or 45 and above (AOR = 0.49, 95% CI = 0.39–0.61) had reduced odds of unintended pregnancies as compared to their counterparts living in poor households, those who are non-users and have no intentions of using the contraceptive, women from the central region and those whose husbands are less than 25 years respectively.

**Table 3:** Direct determinants of unintended pregnancies

Selected characteristic	AOR(95%CI)
Woman’s age	
15–24^†^	1.00
25–34	0.37(0.33–0.43)**
35–49	0.12(0.09–0.15)**
Wealth index	
Poor^†^	1.00
Middle	1.03(0.91–1.16)
Rich	0.72(0.64–0.82)**
Woman’s education level	
No education^†^	1.00
Primary	1.44(1.24–1.67)**
Secondary/Higher	1.28(1.06–1.54)*
Place of residence	
Urban^†^	1.00
Rural	1.16(1.03–1.31)*
Region	
Central^†^	1.00
Eastern	1.25(1.11–1.42)**
Western	0.72(0.63–0.82)**
Northern	1.46(1.26–1.69)**
Religion	
Catholic^†^	1.00
Anglican	1.06(0.95–1.17)
Muslim	1.33(1.16–1.53)**
Others	1.21(1.07–1.38)**
Occupation	
Not working^†^	1.00
Professional/technical/managerial/ clerical	0.98(0.81–1.20)
Agricultural/household and domestic	0.99(0.87–1.12)
Sales/services	1.01(0.86–1.19)
Manual	0.96(0.82–1.12)
Access to family planning messages	
No^†^	1.00
Yes	1.03(0.93–1.13)
Husband’s age	
≤24^†^	1.00
25–34	0.86(0.72–1.01)
35–44	0.73(0.60–0.89)**
45 and above	0.49(0.39–0.61)**
Husband’s education level	
No education^†^	1.00
Primary	1.47(1.21–1.79)**
Secondary/Higher	1.48(1.20–1.82)**
Do not know	1.52(1.10–2.11)*
Knowledge on ovulation	
During/after period^†^	1.00
Middle of the cycle	0.95(0.86-–.06)
Before period begins	1.17(1.00–1.36)
Do not know	1.28(1.14–1.45)**
Contraceptive use and intention	
No^†^	1.00
Yes	0.47(0.42–0.54)**
Discontinuation of contraceptives	
No^†^	1.00
Yes	1.74(1.58–1.92)**
Age at sexual debut	1.54(1.04–2.28)*
Age at first marriage	0.95(0.71–1.26)
Age at first birth	3.36(2.19–5.16)**
Total children ever born	1.42(1.38–1.46)**

† is a reference category; AOR is the adjusted odds ratio; 95% CI is the confidence interval at 95%; *p < 0.05, **p < 0.01; the assessment was based generalized structural equation model with χ^2^<0.001

Surprisingly married women with primary education (AOR = 1.44, 95% CI = 1.24–1.67) and those with secondary/higher education (AOR = 1.28, 95% CI = 1.06–1.54) had a higher likelihood of unintended pregnancy as compared with those with no education as shown in Table 3. Also, married women whose husbands had primary level of education (AOR = 1.47, 95% CI = 1.21–1.79), secondary/higher education (AOR = 1.48, 95% CI = 1.20–1.82), and those who did not know their husbands level of education (AOR = 1.52, 95% CI = 1.10–2.11) were significantly associated with increased odds of unintended pregnancy. Further, rural women (AOR = 1.16, 95% CI = 1.03–1.31), contraceptive discontinuation (AOR = 1.74, 95 %CI = 1.58–1.92), women from the eastern (AOR = 1.25, 95% CI = 1.11–1.42) and northern region (AOR = 1.46, 95%CI = 1.26–1.69), those who do not know about ovulation (AOR = 1.28, 95% CI = 1.14–1.45), and Muslim women (AOR = 1.33, 95% CI = 1.16–1.53) were more likely to experience unintended pregnancies. Results also show that an increase in age at sexual debut (AOR = 1.54, 95% CI = 1.04–2.28), increase in age at first birth (AOR = 3.36, 95% CI = 2.19–5.16), and a higher number of children ever born (AOR = 1.42, 95% CI = 1.38–1.46) were significantly associated with increased odds of unintended pregnancies.

### Indirect determinants of unintended pregnancies among married women

The results in Table 4 revealed that married women with higher age at sexual debut and aged 25–34 years (AOR = 1.03, 95% CI = 1.02–1.03), aged 35–49 (AOR = 1.03, 95% CI = 1.02–1.04), living in rich households (AOR = 1.01, 95% CI = 1.00–1.02), with a primary level of education (AOR = 1.03, 95% CI = 1.02–1.04), secondary/higher education (AOR = 1.11, 95% CI = 1.10–1.13), from western (AOR = 1.02, 95% CI = 1.01–1.03), and northern region (AOR = 1.11, 95% CI = 1.10–1.13) had increased odds of unintended pregnancies. Compared with those who were not working, married women in professional/technical/managerial/clerical (AOR = 1.06, 95% CI = 1.05–1.07), agricultural/household and domestic (AOR = 1.02, 95% CI = 1.01–1.03), sales/services (AOR = 1.02, 95% CI = 1.01–1.03), and manual (AOR = 1.03, 95% CI = 1.02–1.04) work with higher age at sexual debut were at a higher risk of unintended pregnancies.

**Table 4:** Indirect determinants of unintended pregnancies through age at sexual debut and age at first birth

Selected characteristic	Age at sexual debut	Age at first birth
	AOR(95%CI)	AOR(95%CI)
Woman’s age		
15–24^†^	1.00	1.00
25–34	1.03(1.02–1.03)**	1.03(1.02–1.03)**
35–49	1.03(1.02–1.04)**	1.04(1.03–1.05)**
Wealth index		
Poor^†^	1.00	1.00
Middle	1.00(0.99–1.01)	0.99(0.99–1.00)
Rich	1.01(1.00–1.02)**	0.99(0.99–1.00)
Woman’s education level		
No education^†^	1.00	1.00
Primary	1.03(1.02–1.04)**	1.01(1.00–1.02)*
Secondary/Higher	1.11(1.10–1.13)**	1.06(1.05–1.07)**
Place of residence		
Urban^†^	1.00	1.00
Rural	0.99(0.99–1.00)*	1.00(0.99–1.00)
Region		
Central^†^	1.00	1.00
Eastern	0.97(0.96–0.97)**	0.98(0.98–0.99)**
Western	1.02(1.01–1.03)**	1.02(1.02–1.03)**
Northern	1.02(1.01–1.03)**	1.01(1.00–1.02)*
Religion		
Catholic^†^	1.00	1.00
Anglican	0.99(0.98–0.99)**	0.99(0.99–1.00)*
Muslim	0.97(0.96–0.98)**	0.99(0.98–0.99)**
Others	1.00(0.99–1.01)	1.00(0.99–1.00)
Occupation		
Not working^†^	1.00	1.00
Professional/technical/managerial/clerical	1.06(1.05–1.07)**	1.03(1.02–1.04)**
agricultural/household and domestic	1.02(1.01–1.03)**	1.00(1.00–1.01)
Sales/services	1.02(1.01–1.03)**	1.00(0.99–1.01)
Manual	1.03(1.02–1.04)**	1.00(0.99–1.01)
Access to family planning messages		
No^†^	1.00	1.00
Yes	1.00(1.00–1.01)	1.00(0.99–1.00)
Husband’s age		
≤24^†^	1.00	1.00
25–34	0.99(0.98–1.01)	1.00(0.99–1.01)
35–44	0.98(0.96–0.99)**	0.98(0.97–0.99)**
45 and above	0.97(0.96–0.99)**	0.98(0.97–0.99)**
Husband’s education level		
No education^†^	1.00	1.00
Primary	0.96(0.95–0.98)**	0.98(0.97–0.99)**
Secondary/higher	0.99(0.97–1.00)*	1.00(0.99–1.01)
Do not know	0.97(0.95–0.99)**	0.96(0.94–0.98)**
Age at first marriage	**–**	1.63(1.61–1.65)**

† is a reference category; AOR is the adjusted odds ratio; 95% CI is the confidence interval at 95%; *p < 0.05, **p < 0.01; the assessment was based generalized structural equation model with χ^2^<0.001

On contrary, married women with a higher age at sexual debut and were Anglican (AOR = 0.99, 95% CI = 0.98–0.99), Muslim (AOR = 0.97, 95% CI = 0.96–0.98), from eastern region (AOR = 0.97, 95% CI = 0.96–0.97), with husbands aged 35–44 (AOR = 0.98, 95% CI = 0.96–0.99) or 45 and above years (AOR = 0.97, 95% CI = 0.96–0.99) had reduced odds of unintended pregnancies. Still, compared with married women whose husbands had no education, those whose spouses had primary education (AOR = 0.96, 95% CI = 0.95–0.98), and secondary education (AOR = 0.99, 95% CI = 0.97–1.00) were less likely to experience unintended pregnancies.

In relation to age at first birth, unintended pregnancies were significant with age at first marriage through age at first birth (AOR = 1.63, 95% CI = 1.61–1.65). Further, women with higher age at sexual debut and aged 25–34 years (AOR = 1.03, 95% CI = 1.02–1.03), aged 35–49 (AOR = 1.04, 95% CI = 1.03–1.05), with primary level of education (AOR = 1.01, 95% CI = 1.00–1.02), secondary/higher education (AOR = 1.06, 95% CI = 1.05–1.07), employed in professional/technical/managerial/clerical (AOR = 1.03, 95% CI = 1.02–1.04), from western (AOR = 1.02, 95% CI = 1.02–1.03), and northern region (AOR = 1.01, 95% CI = 1.00–1.02) had increased odds of unintended pregnancies. However, married women with a higher age at the age at first birth and were Anglican (AOR = 0.99, 95% CI = 0.99–1.00), Muslim (AOR = 0.99, 95% CI = 0.98–0.99), from eastern region (AOR = 0.98, 95% CI = 0.98–0.99), with husbands aged 35–44 (AOR = 0.98, 95% CI = 0.97–0.99) or 45 and above years (AOR = 0.98, 95% CI = 0.97–0.99), and with husbands with primary education (AOR = 0.98, 95% CI = 0.97–0.99) had reduced odds of unintended pregnancies.

The findings in Table 5 show the indirect determinants of unintended pregnancies through children ever born and knowledge on ovulation. Married women aged 25–34 who had more children (AOR = 1.87, 95% CI = 1.80–1.94) and those aged 35–49 years with the same number of children (AOR = 2.79, 95% CI = 2.68–2.91) were significantly associated with increased risk of unintended pregnancies. Relatedly, married women with a higher number of children and were in rural areas (AOR = 1.08, 95% CI = 1.05–1.11), in the eastern region (AOR = 1.04, 95% CI = 1.01-1.07), Muslim (AOR = 1.04, 95% CI = 1.00–1.07) and with husbands aged 25–34, 35–44 and more than 44 years (AOR = 1.35, 95% CI = 1.27–1.44; AOR = 1.59, 95% CI = 1.49–1.70; (AOR = 1.71, 95% CI = 1.60–1.83) were more likely to have unintended pregnancies those in urban areas, central region, Catholics, and those with husbands below 25 years. On contrary, married women with more children and were from rich households (AOR = 0.94, 95% CI = 0.91–0.96), with primary education (AOR = 0.94, 95% CI = 0.92–0.97), secondary/higher education (AOR = 0.82, 95% CI = 0.79–0.85), northern region (AOR = 0.96, 95% CI = 0.93–1.00), employed in professional/technical/managerial/clerical (AOR = 0.93, 95% CI = 0.89–0.97) or sales/services (AOR = 0.95, 95% CI = 0.92–0.99), had access to family planning messages through media (AOR = 0.97, 95%CI = 0.95–0.99), whose husbands had secondary/higher education level (AOR = 0.91, 95% CI = 0.87–0.94) and higher age at first birth (AOR = 0.43, 95% CI = 0.40–0.45) were significantly associated with reduced risk of unintended pregnancy.

**Table 5:** Indirect determinants of unintended pregnancies through children ever born and knowledge about ovulation

Selected characteristic	Children ever born	No knowledge on ovulation
	AOR(95%CI)	AOR(95%CI)
Woman’s age		
15–24^†^	1.00	1.00
25–34	1.87(1.80–1.94)**	0.92(0.78–1.08)
35–49	2.79(2.68–2.91)**	0.94(0.74–1.19)
Wealth index		
Poor^†^	1.00	1.00
Middle	1.00(0.97–1.02)	0.82(0.70–0.96)*
Rich	0.94(0.91–0.96)**	0.83(0.71–0.98)*
Woman’s education level		
No education^†^	1.00	1.00
Primary	0.94(0.92–0.97)**	0.77(0.65–0.91)*
Secondary/Higher	0.82(0.79–0.85)**	0.58(0.46–0.72)*
Place of residence		
Urban^†^	1.00	1.00
Rural	1.08(1.05–1.11)**	1.02(0.87–1.18)
Region		
Central^†^	1.00	1.00
Eastern	1.04(1.01–1.07)**	0.74(0.63–0.86)**
Western	0.97(0.95–1.00)	0.71(0.60–0.83)**
Northern	0.96(0.93–1.00)*	0.53(0.44–0.64)**
Religion		
Catholic^†^	1.00	1.00
Anglican	1.02(0.99–1.04)	1.00(0.88–1.14)
Muslim	1.04(1.00–1.07)*	0.99(0.83–1.18)
Others	1.05(1.02–1.08)**	1.11(0.95–1.31)
Occupation		
Not working^†^	1.00	1.00
Professional/technical/managerial/ clerical	0.93(0.89–0.97)**	1.14(0.89–1.46)
agricultural/household and domestic	1.03(1.00–1.06)	0.79(0.68–0.93)**
Sales/services	0.95(0.92–0.99)*	0.83(0.68–1.02)
Manual	0.98(0.95–1.02)	0.78(0.64–0.94)*
Access to family planning messages		
No^†^	1.00	1.00
Yes	0.97(0.95–0.99)**	0.76(0.68–0.86)**
Husband’s age		
≤24^†^	1.00	1.00
25–34	1.35(1.27–1.44)**	0.89(0.73–1.09)
35–44	1.59(1.49–1.70)**	0.78(0.61–1.00)*
45 and above	1.71(1.60–1.83)**	0.92(0.70–1.21)
Husband’s education level		
No education^†^	1.00	1.00
Primary	0.98(0.95–1.02)	1.22(0.97–1.53)
Secondary/Higher	0.91(0.87–0.94)**	1.16(0.90–1.48)
Do not know	0.90(0.84–0.96)**	2.28(1.62–3.23)**
Age at first birth	0.43(0.40–0.45)**	
Total children ever born	**–**	0.99(0.96-1.02)

† is a reference category; AOR is the adjusted odds ratio; 95% CI is the confidence interval at 95%; *p < 0.05, **p < 0.01; the assessment was based generalized structural equation model with χ^2^<0.001

Further, as compared with the poor, those with no education, being unemployed, having no access to family planning messages through media, staying in the central region and those with husbands below 25 years, married women who did not have knowledge on ovulation and were from the middle (AOR = 0.82, 95% CI = 0.70–0.96) or rich (AOR = 0.83, 95% CI = 0.71–0.98) households, with primary (AOR = 0.77, 95% CI = 0.65–0.91) or secondary/higher (AOR = 0.58, 95% CI = 0.46–0.72), employed in agricultural/household and domestic work (AOR = 0.79, 95% CI = 0.68–1.02) or manual work (AOR = 0.78, 95% CI = 0.64–0.94), had access to family planning messages (AOR = 0.76, 95% CI = 0.68–0.86), staying in eastern (AOR = 0.74, 95% CI = 0.63–0.86), western (AOR = 0.71, 95% CI = 0.60–0.83) and northern region (AOR = 0.53, 95% CI = 0.44–0.64), and those with husbands aged 35–44 years (AOR = 0.78, 95% CI = 0.61–1.00) were less likely to have unintended pregnancies. Also, married women who did not both their husband’s level of education and knowledge on ovulation were significantly associated with a higher risk of unintended pregnancies (AOR = 2.28, 95% CI = 1.62–3.23).

Results in Table 6 show the determinants of unintended pregnancies through contraceptive use and intention and discontinuation of contraceptives. Married women aged 35–44 years who were using and had intentions of contraceptives (AOR = 2.21, 95% CI = 1.83–2.69) and those aged 25–34 years (AOR = 1.25, 95% CI = 1.10–1.41) who had discontinued the use of contraceptives were more likely to have unintended pregnancies as compared with their counterparts below 25 years. Relatedly, married women in eastern (AOR = 1.22, 95% CI = 1.06–1.42), western (AOR = 1.47, 95% CI = 1.27–1.69), those in the northern region (AOR = 1.41, 95% CI = 1.19–1.66), women whose husbands were above 44 years (AOR = 1.50, 95% CI = 1.16–1.93), and those who did not have knowledge on ovulation (AOR = 1.37, 95% CI = 1.20–1.56) were more likely to experience unintended pregnancies. On contrary, married women with primary education (AOR = 0.57, 95% CI = 0.50–0.66), secondary/higher education (AOR = 0.53, 95% CI = 0.44–0.64), Anglican (AOR = 0.86, 95% CI = 0.76–0.96), those in agricultural/household and domestic (AOR = 0.78, 95% CI = 0.67–0.89), sales/services (AOR = 0.65, 95% CI = 0.54–0.79), and manual (AOR = 0.80, 95% CI = 0.68–0.95), women who had access to family planning messages (AOR = 0.85, 95% CI = 0.77–0.95), those whose partners are aged 25–34 years (AOR = 0.81, 95% CI = 0.66–0.99) and women whose husbands had primary education (AOR = 0.55, 95% CI = 0.46–0.65) or secondary/higher education (AOR = 0.54, 95% CI = 0.45–0.66) provided were using or had the intention of contraceptives were associated with reduced risks of unintended pregnancies.

**Table 6:** Indirect determinants of unintended pregnancies through contraceptive use and intention and discontinuation of contraceptives

Selected characteristic	Contraceptive use and intention	Discontinuation of contraceptives
	AOR(95%CI)	AOR(95%CI)
Woman’s age		
15–24^†^	1.00	1.00
25–34	1.04(0.88–1.22)	1.25(1.10–1.41)**
35–49	2.21(1.83–2.69)**	1.00(0.84–1.19)
Wealth index		
Poor^†^	1.00	1.00
Middle	0.91(0.80–1.05)	1.26(1.11–1.43)**
Rich	0.93(0.81–1.07)	1.13(0.99–1.29)
Woman’s education level		
No education^†^	1.00	1.00
Primary	0.57(0.50–0.66)**	1.39(1.18–1.64)**
Secondary/Higher	0.53(0.44–0.64)**	1.12(0.92–1.36)
Place of residence		
Urban^†^	1.00	1.00
Rural	1.14(1.00–1.31)	0.92(0.81–1.03)
Region		
Central^†^	1.00	1.00
Eastern	1.22(1.06–1.42)**	1.30(1.14–1.48)**
Western	1.47(1.27–1.69)**	1.13(0.99–1.29)
Northern	1.41(1.19–1.66)**	1.08(0.93–1.26)
Religion		
Catholic^†^	1.00	1.00
Anglican	0.86(0.76–0.96)*	1.14(1.03–1.27)*
Muslim	1.08(0.92–1.27)	1.21(1.05–1.39)**
Others	1.15(1.00–1.33)*	1.04(0.91–1.19)
Occupation		
Not working^†^	1.00	1.00
Professional/technical/managerial/ clerical	0.81(0.65–1.01)	0.86(0.70–1.04)
agricultural/household and domestic	0.78(0.67–0.89)**	0.91(0.79–1.04)
Sales/services	0.65(0.54–0.79)**	1.02(0.86–1.19)
Manual	0.80(0.68–0.95)*	0.99(0.84–1.16)
Access to family planning messages		
No^†^	1.00	1.00
Yes	0.85(0.77–0.95)**	1.14(1.03–1.26)*
Husband’s age		
≤24^†^	1.00	1.00
25–34	0.81(0.66–0.99)*	1.59(1.32–1.92)**
35–44	0.91(0.71–1.15)	1.74(1.40–2.15)**
45 and above	1.50(1.16–1.93)**	1.41(1.11–1.79)**
Husband’s education level		
No education^†^	1.00	1.00
Primary	0.55(0.46–0.65)**	1.26(1.01–1.56)*
Secondary/Higher	0.54(0.45–0.66)**	1.34(1.06–1.68)*
Do not know	0.53(0.37–0.74)**	1.23(0.87–1.73)
Knowledge on ovulation		
During/after period^†^	1.00	
Middle of the cycle	1.01(0.90–1.14)	
Before period begins	0.94(0.78–1.12)	
Don’t know	1.37(1.20–1.56)**	
Contraceptive use and intention		
No^†^		1.00
Yes		0.47(0.41–0.53)**

† is a reference category; AOR is the adjusted odds ratio; 95% CI is the confidence interval at 95%; *p < 0.05, **p < 0.01; the assessment was based generalized structural equation model with χ^2^<0.001

In relation to contraceptive discontinuation, married women who had discontinued the use of contraceptives and were living in the middle wealth quintile (AOR = 1.26, 95% CI = 1.11–1.43), had primary education (AOR = 1.39, 95% CI = 1.18–1.64), living in the eastern region (AOR = 1.30, 95% CI = 1.14–1.48), Anglican (AOR = 1.14, 95% CI = 1.03–1.27), Muslims (AOR = 1.21, 95% CI = 1.05–1.39), had access to family planning messages (AOR = 1.14, 95% CI = 1.03–1.26) were more likely to have unintended pregnancies. Similarly, married women whose husbands were aged 25–34 years (AOR = 1.59, 95% CI = 1.32–1.92 or 35–44 years (AOR = 1.74, 95% CI = 1.40–2.15) or above 44 years (AOR = 1.41, 95% CI = 1.11–1.79), women whose husbands had primary education (AOR = 1.26, 95% CI = 1.01–1.59), secondary/higher education (AOR = 1.34, 95% CI = 1.06–1.68) and had discontinued the use of contraceptives were associated with increased risk of unintended pregnancies. However, married women who had discontinued contraceptives but had the intention of using contraceptives were less likely to have unintended pregnancies (AOR = 0.47, 95% CI = 0.41–0.53). Table 7 presents the regression diagnosis of the models that were used in the study. Model 3 (controlling for woman and husband’s demographic and socioeconomic factors and the intermediate factors simultaneously) was selected for the final analysis as it showed the best fit for the data.

**Table 7:** Regression diagnostics of the models

	Log likelihood	AIC	BIC
Model 1	−117913	235,924	236,283
Model 2	−118,683	237,494	237,962
Model 3	−33,349	67,242	69,232

## Discussion

The main objective of the study was to explore the direct and indirect determinants of unintended pregnancies among married women in Uganda using the generalized structural equation model. Results showed overall, 37% of the pregnancies were unintended. Woman’s age, religion, wealth index, occupation, woman’s education level, region, place of residence, husband’s age, husband’s education level, age at first sex, age at first marriage, age at first birth, children ever born, knowledge on ovulation, contraceptive use and intention, and discontinuation of contraceptives either directly or indirectly impacted on unintended pregnancies.

Findings show that older women were less likely to have unintended pregnancies as compared with their young counterparts. This was consistent with earlier findings in India [32] and Malawi [26]. The reason for this could be that older women are assumed to have more in-depth knowledge and are informed about the benefits of contraceptives. Also, young women may be reluctant and fear to openly discuss family planning issues with their partners or even seek guidance from health personnel. However, results also showed that older women who had taken long to have their first birth, or to initiate sex, had a higher number of children, and had discontinued the use of contraceptives had an increased risk of unintended pregnancies.

In this study, we found that married women from the highest wealth quintile were at a lower risk of experiencing unintended pregnancies as compared with those in the lowest wealth quintile. This was in agreement with prior studies [23, 33, 34] elsewhere. Still, women with better economic status who initiated sex at a later age or discontinued the use of contraceptives were more likely to have unintended pregnancies. However, those who did not have knowledge about ovulation or had more children but were from rich households were significantly associated with reduced risk of unintended pregnancies. The reason for this could be that women from wealthier households are likely to be empowered with information on how to regulate fertility and have access to family planning services therefore able to have their desired number of children.

Surprisingly, results revealed that educated women and those whose husbands were educated were more likely to have unintended pregnancies as compared with women who or their husbands were illiterates contrary to other findings [23, 27]. Still, educated women with higher age at sexual debut or age at first birth or a higher number of children born were more likely to have unintended pregnancies. However, women whose husbands were educated despite having a higher age at sexual debut or age at first birth or children born were associated with reduced risk of unintended pregnancies. Additionally, educated women and those whose husbands were also educated and were either using contraceptives or had the intention for future use of contraceptives were less likely to have unintended pregnancy while there was an increased risk through contraceptive discontinuation.

Regarding the place of residence, married women in rural areas were more likely to have unintended pregnancies as compared with their counterparts in urban areas. This was in line with different findings [35]. This could be attributed to affordability and easy access to modern contraceptives among women in urban areas. Also, the study reveals that married women in rural areas who had more children were more likely to report unintended pregnancies as compared with those in urban places. However, postponing age at sexual debut among women in rural areas significantly reduces the risk of unintended pregnancies.

The study still indicated that the high risk of unintended pregnancies was driven by women from the Eastern and Northern regions as compared with other regions. This finding is in line with earlier findings [8]. This could be attributed to the disparities in infrastructural developments in the regions making access to better health services a problem. Surprising, contraceptive use and intention were significantly associated with a high risk of unintended pregnancies among women in all the regions. This needs further qualitative research to explain this behavior. However, postponing age at sexual debut or age at first birth and having knowledge on ovulation among women in the eastern region reduces the risk.

Further, Muslim women were at a higher risk of unintended pregnancies as compared with the Catholics concurring with findings in Bangladesh [23]. Also, having more children and discontinuation of contraceptives among Muslim women puts them at a higher risk of unintended pregnancies. Additionally, having a higher age at sexual debut and at first birth reduces the probability of unintended pregnancies not only among Muslims but also for Anglican women.

Unlike Kenya [25] where employed women are associated with reduced odds of unintended pregnancies, the occupation was not a direct factor in Uganda. Results of the indirect effect though indicate that employed married women with higher age at sexual debut and higher age at first birth were more likely to have unintended pregnancies. However, the risk was reduced through the children ever born and the use of contraceptives or the intention for future use. Employed women are empowered in making decisions regarding their preferred family size including the plan to spacing the births.

Relatedly, access to family planning messages through media did not directly impact unintended pregnancies contradicting earlier studies [36]. The indirect effect revealed that women who had access to family planning messages and had more children or were using contraceptives were less likely to report unintended pregnancies despite lacking knowledge on ovulation. However, discontinuation of contraceptive use while accessing media was positively associated with the risk of unintended pregnancies. Media plays a vital role in educating women on the importance of various modern contraceptive methods including the approaches to accessing each method.

As the age of the husband increased, the intention of a woman to have unintended pregnancies reduced. Different studies have shown that women married to older men are exposed to increased sexual risk behaviors [37] including unplanned pregnancies which are contrary to this finding. Our finding further indicated that older men whose wives had higher age at sexual debut, higher age at first birth, and intention or use of contraceptives were significantly associated with reduced odds of unintended pregnancies while discontinuation of contraceptives being associated with increased risk. This finding indicates older men could have reached their desired family sizes and therefore opt for measures to stop further pregnancies which may not be the case with younger men.

Unintended pregnancy also showed a strong relation with deficient knowledge on ovulation in this study concurring with previous studies [33]. Findings also show that women who did not have knowledge on ovulation and were using contraceptives were more likely to have unintended pregnancies. This confirms that the high rate of contraceptive failure among women. There is a need for sex education and information on family planning.

In regard to contraceptive use and intention, married women who were using contraception or were not using but had the intention for future use were less likely to register unintended pregnancies. This was in agreement with studies in Ethiopia [34]. Also, contraceptive use and intention were negatively associated with unintended pregnancies through discontinuation of contraceptives. Further, married women who had discontinued contraceptives were more likely to have unintended pregnancies compared with those who were using contraceptives. Reasons for discontinuation or non-use of contraceptives include high costs, fear of side effects, cultural, or partner-related factors [38]. Therefore, efforts should be made to provide quality contraceptive counseling, informing women about side effects, having a variety of contraceptive methods to choose from, and making them accessible and affordable will help to decrease contraceptive discontinuation, increase the uptake of contraceptives, and thus reduce unintended pregnancy.

High parity women were positively associated with unintended pregnancies concurring with findings in Ivory Coast [27] and the Philippines [39]. This may be that high parity women may have limited knowledge and access to family planning services, therefore experience difficulty in practicing particular contraceptives.

The results also revealed that postponing age at sexual debut and age at first birth had a positive impact on the risk of unintended pregnancies. Similarly, raising the age at first marriage increases the odds of unintended pregnancies through age at first birth. Mutumba and colleagues showed that women who had a higher age at sexual debut or age at first birth or age at first marriage were less likely to use modern contraceptives [40] while making them prone to unintended pregnancies.

### Limitations

The data used was cross-sectional and therefore was limited in providing an understanding of the timing of unintended pregnancy. Women’s perception on whether the pregnancy was planned or wanted can change over time. Pregnancy intention asked in the early stage of pregnancy is more likely to give an accurate answer than those at the late stage of pregnancy or even after delivering and the different cases are not reflected in the survey. Despite the above limitation, reliable data and appropriate method were used hence the findings reflect accurately the determinants of unintended pregnancies among currently married women in Uganda. The large sample size of this study and its likely representativeness was a great strength as well.

## Conclusion

The study showed that 37% of pregnancies among married women were unintended. Young women, those living in poor households, being educated, staying in rural areas, those in the eastern and northern region, Muslim women, lack of knowledge on ovulation, discontinuation of contraceptives, non-use of and intention for contraceptives, high age at sexual debut, high age at first birth, and high parity were directly associated with a higher risk of unintended pregnancies. Relatedly, discontinuation of contraceptives regardless of the place of residence or region, whether a woman is older/educated or married to an older/educated husband, stays in a wealthier household or could access family planning messages was associated with higher odds of unintended pregnancies. Still, older women and those in rural areas who had more children were more likely to have unintended pregnancies. However, having more children while educated or living in a wealthier household or being able to access family planning messages significantly lowered the risk. Women who are employed, educated, married to educated partners, could access family planning messages, and were either using or had the intention for contraceptives were less likely to report unintended pregnancies.

There is a need to provide quality contraceptive counseling through outreaches so that women are informed about the different contraceptive methods, the possible side effects, having a variety of contraceptive methods to choose from and making them accessible and affordable will not only make them make informed choices but also reduce contraceptive discontinuation. This in turn will increase the uptake of contraceptives and significantly reduce unintended pregnancies. Also, empowering women through job creation and extension of higher education to all people will help couples appreciate the benefits of a small desired family size, be able to provide needs for their families thus reducing risks of unintended pregnancies.

## Data Availability

The datasets used for this study are available from the demographic health and survey website upon request using http://dhsprogram.com/data/.

## Abbreviations

AOR: Adjusted odds ratios
AIC: Akaike Information Criterion
BIC: Bayesian Information Criterion
CI: Confidence interval
DHS: Uganda Demographic and Health Surveys
GSEM: Generalized Structural Equation Model
MoH: Ministry of Health
UBOS: Uganda Bureau of Statistics
UDHS: Uganda Demographic and Health Surveys
VIF: Variance Inflation Factor
